# Cancer immunotherapy and immunomonitoring approached as a future therapy for long-lasting outcomes: outlines of 8th CITIM meeting

**DOI:** 10.3389/fmolb.2025.1660726

**Published:** 2025-10-10

**Authors:** Rostyslav Bilyy

**Affiliations:** Institute of Cellular Biology and Pathology “Nicolae Simionescu”, Bucharest, Romania

**Keywords:** cancer, immunomonitoring, CITIM, immunotherapy, conference

## 1 Introduction

Cancer Immunotherapy and Immunomonitoring (CITIM) turned sixteen in Bucharest, where the eighth International Conference was held from March 30 to 4 April 2025. The event remained true to CITIM’s credo: advancing the frontiers of cancer immunotherapy and immunomonitoring in Eastern European countries by bringing together world leaders in the field and sharing knowledge with the next-generation of researchers.

Romania was chosen as the venue due to its recent strides in integrating cancer immunotherapy into clinical practice, with several thousand successful patient cases already recorded. The meeting, organized by CITIM President Prof. Rostyslav Bilyy under the auspices of two leading Romanian research institutions—The Institute of Cellular Biology and Pathology “Nicolae Simionescu” and The Romanian Society of Immunology—delivered CITIM’s boldest scientific programme to date.

Over 4 days, more than 100 participants engaged in 10 scientific sessions, four plenary lectures, and over 20 invited talks, all centered on one revolutionary message: next-generation immunotherapy must integrate neuroimmune signaling, metabolic rewiring, myeloid complexity, neutrophil dynamics, and advanced antigen-delivery technologies. The future lies in the intelligent combination of immunotherapy with radiation, chemotherapeutics, and vaccines to achieve more precise and effective cancer treatments.

Below is a summary of key perspectives and findings in cancer immunotherapy and immunomonitoring, based on research presented at CITIM-2025.

## 2 Neuro-metabolic-immune regulation: expanding the mind-body-cancer connection

The integration of neuroscience and immunology, first introduced at the inaugural CITIM meeting (Umansky et al., 2009) in 2009, reached an unprecedented level of sophistication at CITIM-2025. Speakers presented compelling evidence that cancer progression is driven by complex, bidirectional communication between the nervous and immune systems. This emerging interdisciplinary field holds the potential to revolutionize our understanding of how psychological stress, autonomic dysregulation, and neuroinflammation contribute to tumor development and resistance to therapy.

A highlight of the conference was the plenary lecture by Prof. Tak W. Mak, which offered a conceptual framework linking metabolic mutations with neuronal mediators that directly influence T cell fate. His group’s research demonstrated that isocitrate dehydrogenase mutations—prevalent in gliomas and acute myeloid leukemia—lead to the production of the oncometabolite 2-hydroxyglutarate (Gross et al., 2010), which profoundly alters both cellular metabolism and the epigenetic landscape. Even more remarkably, his team revealed recent findings that neurotransmitters such as acetylcholine and norepinephrine act as direct immunomodulators, affecting T cell differentiation, activation, and memory formation via specific receptor-mediated pathways. These findings suggest critical roles for neuroimmune interactions not only in infection and autoimmunity but also in cancer initiation and progression (Mak, 2025).

Prof. Michael Shurin, one of the co-founders of CITIM, presented a comprehensive analysis of Schwann cell biology, positioning these peripheral nervous system cells as key orchestrators of tumor immune evasion. Traditionally known for their roles in nerve function and regeneration, Schwann cells have now emerged as active players in shaping immunosuppressive tumor microenvironments. Upon infiltrating tumors, Schwann cells undergo phenotypic reprogramming that enhances their capacity to recruit and activate myeloid-derived suppressor cells (MDSCs), regulatory dendritic cells, and regulatory T cells (Tregs), while simultaneously promoting tumor cell invasiveness. Importantly, Schwann cell-derived exosomes enriched in microRNA-21-5p were shown to directly target tumor cells, leading to the upregulation of genes associated with epithelial-mesenchymal transition and increased metastatic potential (Shurin, 2025). The contact dependent and independent effects in interactions between Schwann cells and metastatic breast carcinoma was reported by Nuray Erin (Erin, 2025).

Jonathan Weiss’s work on metabolic reprogramming revealed how targeting specific metabolic pathways could overcome immunosuppression in the tumor microenvironment. His research focused on itaconate, a metabolite highly upregulated in tumor-associated macrophages, which promotes fatty acid oxidation and mitochondrial reactive oxygen species generation that facilitate tumor growth (Weiss et al., 2025). While Luca Vannucci’s work focused on targeting the tumor immune microenvironment with nano-therapies and patient-tailored treatments, highlighting how interactions between cancer cells, tissue components, and the immune system determine tumor evolution and influence treatment efficacy (Vannucci et al., 2025).

Opinion: The evidence points toward cancer as a neuro-immune-metabolic syndrome requiring integrated therapeutic approaches.

## 3 Chronic inflammation and tumor tolerance are smoldering fires that ignite malignant growth

Chronic inflammation greatly contributes to the relocation of immune system’s attention and allowing the tumors to escape from strict control, while continuous exposure to tumor leads to tumor tolerance. Tolerance reversal—once relegated to autoimmune disease research—emerged as a central theme throughout CITIM-2025, with speakers demonstrating how tumors systematically delete, subvert, and rewire immune recognition mechanisms. The complexity extends far beyond simple antigen presentation defects, encompassing sophisticated cellular networks that actively maintain immune ignorance. The idea that tumor is already tolerated by the host, and the only way to treat cancer is to break the tolerance was proposed in lectures of M. Herrmann, R. Alon, M. Elkabetz.

Prof. Adit Ben-Baruch’s presentation on TNFα networks revealed the tight connection between chronic inflammation and cancers, particularly in triple-negative breast cancer. Her work demonstrated that continuous TNFα presence creates chronic tumor inflammation that fundamentally alters tumor growth and metastasis potential. Unlike TNFR1, which primarily mediates pro-inflammatory responses, TNFR2 showed context-dependent effects. In triple-negative subtype of breast cancer, high TNFR2 expression on tumor-infiltrating lymphocytes (TIL) correlated with improved patient prognosis, while TNFR2+ tumor cells showed reduced metastatic potential. This suggests that TNFR2 preserves anti-tumor immune function even in inflammatory environments, making it an attractive therapeutic target for preserving beneficial immune responses while blocking harmful inflammation. The author proposed that TNBC patients should be treated by TNFR1-specific modalities, while sparing TNFR2 (Ben-Baruch, 2025).

Elena Voronov’s presentation detailed the involvement of tumor cell-associated IL-1α in the progression and metastasis of breast carcinoma in mice. Her work also touches on how the microbiota in colitis influences the crosstalk with MDSCs, which acts as a predisposing factor for colitis-associated colorectal cancer (Machluf-Kaz et al., 2025).

The differences in CD8 and CD4 antigens towards tumor antigens NY-ESO-1, Melan-A, MAGE-A3 and survivin in context of melanoma treatment was reported by Graham Pawelec (Pawelec, 2025). Paul Lehmann and Greg Kirchenbaum, representing CTL - a partner and general sponsor of CITIM, presented on how multiplexed ImmunoSpot assays enable detailed assessment of antigen-specific B cell frequency, class usage, and functional affinity. Complex interplay between dendritic and T-cells in tumor draining lymph nodes was demonstrated by Ronen Alon (Levi et al., 2025). Genome-wide levels of acetylation of the lysin 27 in histone 3 (H3K27ac) positively correlated with immune-related signature indicating inflamed tumor microenvironment and inversely correlated with survival, as reported by Andreas Lundqvist (Cruz De los Santos and Lundqvist, 2025).

Opinion: Strict control of chronic inflammation is a pre-requisite to manage tumor growth, progression and metastases. Reversal of tumor tolerance is a need for effective destruction of tumors.

## 4 Complexity of MDSC cells: unraveling heterogeneity and therapeutic windows

Myeloid-derived suppressor cells (MDSCs) emerged at CITIM-2025 as one of the most complex and therapeutically challenging components of tumor immunology. Rather than representing a uniform immunosuppressive population, MDSCs comprise a highly heterogeneous group of cells with distinct ontogenies, activation states, and functional properties that vary significantly depending on tumor type, anatomical location, and disease stage.

In his plenary lecture, Prof. Viktor Umansky—one of the co-founders of CITIM—outlined two primary mechanisms underlying MDSC generation: impaired myeloid differentiation driven by soluble inflammatory mediators, and the active conversion of mature myeloid cells into suppressive phenotypes. His work in melanoma models demonstrated that tumor-derived extracellular vesicles enriched with HSP90, S100A8/A9, and HMGB1 can directly reprogram mature myeloid cells into functional MDSCs, revealing a previously underappreciated source of immunosuppression (Umansky, 2025).

Prof. Michal Baniyash further expanded this framework by examining MDSC–bacteria interactions in colitis-associated colorectal cancer. Her group showed that MDSCs recruited to chronically inflamed intestinal tissues—such as those seen in inflammatory bowel disease—exhibited enhanced suppressive function. Engulfment of bacteria by these MDSCs activated suppressive gene programs while promoting the release of inflammatory signals that recruited additional MDSCs. This establishes a pathological feedback loop, whereby microbial stimuli sustain both MDSC accumulation and their immunosuppressive activity, ultimately exacerbating tissue disruption and increasing the risk of inflammation-associated carcinogenesis. Targeting this MDSC–microbiota crosstalk was proposed as a strategy to attenuate cancer progression driven by chronic inflammation (Baniyash, 2025).

Opinion: MDSCs are intimately linked to cancer-associated systemic inflammation. Their remarkable functional plasticity positions them as promising targets for reprogramming-based therapies aimed at dismantling tumor-induced immunosuppression.

## 5 Neutrophils and NETs in cancer: a critical frontier in tumor immunity

The role of neutrophils and neutrophil extracellular traps (NETs) in cancer emerged as one of the most dynamic and therapeutically relevant topics at CITIM-2025. Three keynote presentations—by Martin Herrmann, Jadwiga Jablonska, and Zvi Fridlender—collectively established neutrophils as central orchestrators of both tumor progression and immune dysfunction, while simultaneously revealing their potential as therapeutic targets.

Martin Herrmann’s comprehensive analysis of NET formation and function provided the foundational understanding of how these structures contribute to cancer pathogenesis. NETs, composed of decondensed chromatin decorated with histones and antimicrobial proteins, were originally discovered as host defense mechanisms against pathogens. However, Herrmann’s work revealed their dark side in cancer biology. NETs display multiple tumor-modifying features that fundamentally alter cancer progression. They create “sticky” scaffolds in the bloodstream that promote metastatic cell adhesion and growth, while simultaneously degrading extracellular matrix components to facilitate tumor invasion. Perhaps most concerning, NETs stimulate neo-angiogenesis, shield cancer cells from immune attack, and promote immunosuppressive environments that favor tumor progression. Crucially, Herrmann demonstrated that NET aggregates, formed in high neutrophil density environments, can persist for months or years while accumulating additional inflammatory mediators like complement and fibrin. In certain locations, these aggregates undergo calcification, creating chronic inflammatory foci that continuously stimulate tumor progression. This persistence explains why neutrophil infiltration correlates with poor prognosis across multiple cancer types (Dölling et al., 2025).

Jadwiga Jablonska’s pioneering work on neutrophil-specific STAT3 targeting revealed the therapeutic potential of reprogramming neutrophils from tumor-promoting to tumor-suppressing phenotypes. Her research demonstrated that STAT3 signaling in neutrophils drives their acquisition of immunosuppressive functions, and that selective STAT3 inhibition could reverse this process (Jablonska, 2025).

Zvi Fridlender’s investigation of NET-immune cell interactions revealed unexpected complexity in neutrophil-mediated immune regulation. His team discovered that neutrophils from lung cancer patients produce smaller amounts of NETs compared to healthy donors, but these cancer-associated NETs retain potent biological activities that influence both tumor cells and immune responses. NETs enhanced T cell activation through mechanisms that were partially DNA-dependent for CD8 T cells. Involvement of additional metabolic pathways for CD4 and CD8 T cells was reported suggesting that NETs function as complex signaling platforms that integrate multiple activation pathways (Al-Sharif et al., 2025).

Opinion: The evidence supports the relevance of neutrophil-targeted combination therapies that could include selective STAT3 inhibitors, optimized NET modulators, and neutrophil reprogramming agents.

## 6 Novel approaches to target immunity with radiation and chemicals: engineering immune synergy

The integration of radiation therapy with immunotherapy reached new levels of sophistication at CITIM-2025, with speakers demonstrating that radiation should be viewed not merely as a tumor-killing modality but as a programmable immune stimulus capable of generating antigen, adjuvant, and favorable microenvironmental changes simultaneously.

Udo Gaipl’s lecture established the conceptual framework by demonstrating how radiation dose, dose rate, fractionation, and timing can be precisely calibrated to generate optimal immune responses. His work on FLASH radiotherapy—ultra-high dose rate radiation delivery—revealed that temporal aspects of radiation delivery fundamentally alter immune consequences. FLASH protocols generated strong immunogenic cell death signals while minimizing normal tissue immunosuppression, creating therapeutic windows previously thought impossible (Gaipl, 2025).

Yona Keisari’s presentation on Diffusing Alpha-emitters Radiation Therapy (DaRT) exemplified next-generation approaches to radio-immunotherapy integration. This novel approach delivers radioactive sources directly into tumors, creating controlled ablation zones that generate massive antigen release combined with danger signal production. Unlike external beam radiation, DaRT creates sustained antigen availability over days to weeks, providing prolonged immune stimulation that promotes memory T cell formation (Keisari, 2025).

Klaus Spohr’s innovative work on immunotherapy-supported Boron Neutron Capture Therapy represented a paradigm shift toward immune cell-delivered radiation. His team developed methods to load immunocompetent cells with boron nanoparticles, effectively creating “cellular radiopharmaceuticals” that selectively deliver radiation to tumor sites. This approach combines the targeting specificity of immune cell trafficking with the precision of nuclear medicine (Spohr et al., 2025).

Rostyslav Bilyy’s work on reversible thiol binder demonstrated how chemical agents can be designed to simultaneously kill cancer cells and stimulate immune responses. This compound selectively accumulates in cancer cell lysosomes, increases reactive oxygen species production, and triggers lysosomal disruption leading to immunogenic cell death. The resulting immune activation proved sufficient to generate long-term anti-tumor immunity in animal models (Arkhypov et al., 2025).

Perspective: The field is transitioning from simple combination approaches to engineered immune-radiation/chemical synergy. The goal is creating programmable immune activation were radiation and chemicals function as precision tools for immune system education and activation.

## 7 Novel vaccine approaches: from precision targeting to multi-modal activation

Cancer vaccination strategies presented at CITIM-2025 revealed a sophisticated evolution beyond traditional peptide-based approaches toward complex, multi-component systems designed to simultaneously engage multiple immune pathways while overcoming established tolerance mechanisms.

Angel Porgador’s plenary presentation on current immunotherapy status established the conceptual challenges facing cancer vaccination (Porgador, 2025). Michael Nishimura in his lecture summarized currently used CAR, TCR and TIL therapies, with a special attention paid to CD19 CAR T cells’ use in clinical setting (Nishimura, 2025).

Sjoerd van der Burg’s groundbreaking work on TEIPP vaccination (T cell epitopes associated with impaired peptide processing) exemplified next-generation antigen selection strategies. TEIPP antigens are generated by defective proteasomal processing in cancer cells, creating neo-epitopes that are absent from normal tissues. This approach salvages immunotherapy responses in checkpoint inhibitor-resistant lung cancer by targeting antigens that remain available even when classical MHC class I presentation is compromised. TEIPP vaccination showed efficacy in patients who had failed conventional immunotherapy, suggesting that antigen selection strategies could overcome resistance mechanisms (Emmers et al., 2025).

Flavio Salazar-Onfray’s presentation on TRIMELVax demonstrated how vaccination strategies could harness innate immune system activation to drive adaptive responses. This melanoma vaccine combines heat shock-conditioned cancer cell lysates with mollusk hemocyanin adjuvant, creating a complex antigenic mixture that rapidly induces neutrophil-driven inflammation and subsequent dendritic cell activation (Salazar-Onfray, 2025).

Moshe Elkabets presented research on the development of an AXL/PD-1 Bi-specific-Cell-Engager that targets AXL and PD1 (BiCE AXL/PD1) and increase the interaction between the AXL-expressing tumor cell and CD8^+^ T cells resulting in enhanced anti-tumor lytic activity of the T cells against AXL-expressing tumor cells (Yegodayev and Elkabets, 2025).

Anahid Jewett’s presentation on NK101 supercharged NK cells represented a paradigm shift toward cellular vaccination approaches. Rather than simply providing antigens for T cell recognition, this strategy involves *ex vivo* activation and expansion of natural killer cells that are subsequently reinfused to provide immediate anti-tumor activity while potentially priming adaptive immune responses (Jewett, 2025).

Opinion: The most promising approaches to cancer treatment will likely combine antigen diversity with innate immune activation and tolerance-breaking strategies. Success will require personalized antigen selection guided by individual patient immune profiles and tumor characteristics.

## 8 Progress in tumor immunology and monitoring from Romanian research centers

One of the defining strengths of the CITIM conference lies in its ability to unite internationally renowned scientists with leading local researchers, fostering an environment of cross-border collaboration and mutual inspiration. This year’s meeting exemplified that mission by bringing together representatives from 11 Romanian research institutions, highlighting Romania’s growing leadership in the field of cancer immunotherapy. Experts from key academic and clinical centers—including Bucharest, Cluj-Napoca, Măgurele, and Timişoara—played an active role not only as participants but also as speakers, session chairs, and contributors to high-level scientific discussions. Their strong presence ensured that CITIM-2025 was not only a platform for global exchange but also a catalyst for national capacity-building in cancer immunology.

Livia Sima’s groundbreaking work on tissue transglutaminase (TG2) in ovarian cancer revealed how stromal proteins can function as immunomodulatory targets. Her research demonstrated that TG2 expression in cancer-associated fibroblasts (CAFs) correlates inversely with CD8^+^ T cell infiltration in human ovarian cancer samples. Using TG2 knockout mouse models, her team showed that TG2 deletion in the host significantly reduced tumor burden and increased survival, accompanied by enhanced CD8^+^ T cell infiltration and activation (Sima et al., 2025).

Manuela Banciu’s comprehensive investigation of tumor microenvironment rewiring through lipid nanoparticles demonstrated how nanotechnology approaches could simultaneously target multiple cellular components. Her team developed simvastatin-loaded long-circulating liposomes (LCL-SIM) that showed natural tropism for tumor-associated macrophages while delivering therapeutic payloads to reshape the immune landscape (Patras et al., 2025). While Agata Mlynska, representing previous CITIM host country–Lithuania - reported the deciphering of immune tumor subtypes through profiling of their microenvironment (Mlynska et al., 2025).

Monica Neagu’s work provided insights into genetic and epigenetic traits in cutaneous melanoma, aiming to identify new therapy targets by evaluating genetic alterations in the EGFR-RAS-RAF pathway (Neagu et al., 2025). Their group also explored the impact of adipokines and gut microbiome on melanoma outcomes and the anti-tumor effects of TLR7/8 agonists on NK cells in melanoma models.

Opinion: The CITIM platform has proven to be an exceptional forum for the exchange of ideas between international experts and local institutions leading to long-lasting collaborative projects.

## 9 Perspectives and limitations

Based on the experience gained through the CITIM conferences and numerous discussions with scientists, clinicians, and policymakers from various countries, we have identified key scientific and policy-related barriers that must be addressed to successfully integrate immunomonitoring and cancer immunotherapy into standard clinical practice in developing regions.

Scientific barriers:

a. Lack of local training and expertise. Established research centers in countries with a developed cancer immunotherapy landscape can serve as excellent training hubs. However, they should adopt a structured policy that ensures trained personnel return to their home institutions and continue building local capacity rather than remaining centralized.
b. Limited infrastructure. Insufficient access to advanced equipment and facilities remains a major challenge. This barrier can be partially overcome by consolidating and sharing existing resources among multiple institutions working toward a common goal.
c. Absence of standardized protocols and biobanking. Non-standardized immunomonitoring protocols, lack of harmonized data management, and limited biobanking capacity hinder progress. These challenges can be addressed through structured support and mentoring from countries that have already established these systems. Similar solutions apply to issues related to licensing, access to novel cell lines, and intellectual property (IP) rights.

Policy-related barriers:

a. High costs of immunotherapy. Initially, the cost of treatments such as CAR-T therapy reached approximately USD 1 million per patient. The introduction of point-of-care manufacturing models has significantly reduced costs, with current prices reported at USD 97,000 in Spain and USD 30,000 in Brazil (Hildreth, 2025). While these reductions are encouraging, costs remain a critical barrier for most low- and middle-income countries.
b. Regulations and reimbursement policies. The lack of national treatment guidelines, regulatory frameworks, and state or insurance-based reimbursement mechanisms limits the adoption of immunotherapy. To overcome this barrier, active involvement of decision-making stakeholders from developing countries is essential—a process already initiated within CITIM activities.

Strengthening local research networks is a prerequisite for overcoming both scientific and policy challenges. Such networks should bring together oncologists, immunologists, and healthcare professionals to promote the sustainable development of cancer immunotherapy. A successful example is the coordination initiative in Lithuania, led by CITIM co-organizer Vita Pašukonienė, which demonstrates how collaboration can accelerate progress.

Similar networks are needed in every country. We strongly encourage CITIM participants and all interested parties to build bridges by engaging with local oncology and immunology societies—which are present in most Eastern European countries—and to organize joint events in the coming year. These interactions will reveal numerous shared challenges and opportunities for collaboration. Such initiatives can serve as a starting point for involving policymakers, ultimately addressing the most pressing limitations to implementing immunotherapy in clinical practice.

## 10 Summary

Recent findings clearly demonstrate that immune regulation is a fundamental component of tumor development. Effective cancer treatment is nearly impossible without a comprehensive understanding of the complex interactions between tumors and the immune system—including direct cell-cell contact, metabolic crosstalk, cytokine signaling, and other factors shaping the tumor microenvironment. In his concluding lecture, Prof. Isaac Witz introduced the concept of the “Cancer Ecosystem”, emphasizing the integrated nature of these interactions. He not only summarized key advances in our understanding of cancer–immune system dynamics but also underscored the vast number of unanswered questions that continue to challenge the field. (Witz, 2025).

Recent advances in targeted anti-tumor cell therapies, novel anti-cancer vaccine strategies, and combination treatments with radio- and chemotherapy designed to enhance immunogenicity—along with the development of tumor- and immune-specific delivery systems—have significantly increased our ability to combat cancer more effectively.

Bringing together leading scientists through platforms like CITIM has proven to be a highly effective strategy for advancing knowledge, particularly in countries where cancer immunotherapy and immunomonitoring are only beginning to be integrated into standard clinical practice. As demonstrated by CITIM, such meetings not only raise general awareness of the immune system’s critical role in cancer development but also foster productive, long-term collaborations that often lead to novel and impactful scientific discoveries.
